# Traumatic injuries among Alaska’s young workers: Linking cases from four data systems

**DOI:** 10.1186/s12889-022-14676-7

**Published:** 2023-01-09

**Authors:** Richard Evoy, Laura Syron, Samantha Case, Devin Lucas

**Affiliations:** 1 Western States Division, Centers for Disease Control and Prevention, National Institute for Occupational Safety and Health, 4230 University Dr #310, Anchorage, AK 99508 USA; 2 Western States Division, Centers for Disease Control and Prevention, National Institute for Occupational Safety and Health, Spokane, WA USA

**Keywords:** Workers’ compensation, Trauma registry, Health equity, Occupational safety

## Abstract

**Background:**

Young workers (aged 15–24 years) experience higher rates of job-related injury compared with workers aged 25–44 years in the United States. Young workers may have limited or no prior work experience or safety training, which can contribute to their injury risk. In 2018, Alaska had the second highest work-related fatality rate and 14th highest non-fatal injury rate in the United States. This study aimed to characterize nonfatal and fatal occupational injuries among young workers in Alaska.

**Methods:**

To describe injury patterns among Alaska young workers from 2014–2018, we used data from four datasets: Alaska Workers’ Compensation, Alaska Occupational Injury Surveillance System, Alaska Trauma Registry, and Alaska Fishermen’s Fund. The datasets were merged two at a time and filtered by the worker characteristics (e.g., age and sex) and incident characteristics (e.g., date of injury). Duplicates were then manually identified between the datasets using the variables above. The injury narrative and Occupational Injury and Illness Classification System codes were used last to verify true duplicates. Descriptive analyses were performed after the duplicates were merged.

**Results:**

During the 5-year study period 2014–2018, young workers experienced 20 fatal and 12,886 nonfatal injuries. Residents of Alaska comprised 85% of nonfatal and 70% of fatal injuries. The top three major occupation groups with the highest number of injuries were production (1,391, 14%), food preparation (1,225, 12%), and transportation/material moving (1,166, 11%). The most common events leading to injuries were struck by object or equipment (2,027, 21%), overexertion involving outside sources (1,385, 14%), and struck against object or equipment (905, 9%). The most common nature of injuries were sprains/strains/tears (3,024, 29%), cuts/lacerations (1,955, 19%), and bruises/contusions (1,592, 15%).

**Conclusion:**

Although progress has been made in reducing worker injuries, Alaskan young workers still experience injuries and fatalities frequently. Based on findings, there is a clear need for employers, researchers, public health professionals, parents, and young workers to prioritize young worker safety through an integrated approach, from education and training to adequate workplace supervision and support.

**Supplementary Information:**

The online version contains supplementary material available at 10.1186/s12889-022-14676-7.

## Background

Protecting young workers in Alaska, and the rest of the United States (US), is a pressing occupational safety and health issue. Across the United States, employers have a responsibility to create a safe working environment, but young workers (aged 15–24 years) have higher rates of nonfatal occupational injuries when compared to workers aged 25–44 years [1]. In the United States, young workers represented approximately 12.6% of the US workforce in 2020 [2]. In 2019, the incidence rate of injuries for workers ages 16–19 was 108.2 per 10,000 full-time equivalent (FTE) workers and 96.2 per 10,000 FTE for workers ages 20–24. In 2018, the rate of injuries treated in emergency departments for workers ages 15–19 was 2.2 times greater than the rate for workers 25 years of age and older [3]. Young workers are oftentimes employed in entry level, low paying jobs as seasonal or part-time workers. It is important to highlight the possibility that their lack of experience or confidence might not fully explain the higher rates of nonfatal and fatal occupational injuries. It is possible that other factors, such as young workers participating in more hazardous industries could explain their high injury and fatality rates. If young workers are prone to working in hazardous industries, this could be obscuring the fact that if hazards were removed from these worksites, then the jobs would be safer for everyone, including young workers.

Since the 1980s, government and academic researchers have continuously identified working in Alaska as a public health concern. Many of the reports found that Alaskan workers had an elevated risk of fatal injuries when compared to the rest of the United States. Throughout the 1980s, Alaska workers were seven times more likely to die in a work-related accident than workers in the rest of the United States. Consequently, extensive efforts by government agencies, industry leaders, nongovernmental organizations, and other stakeholders resulted in a significant decrease in the rate of work-related fatalities [4]. However, work-related injuries continue to occur at a relatively high rate. In 2020, Alaska had the 2^nd^ highest work-related fatality rate in the United States with 10.7 fatalities per 100,000 FTEs while the US average was 4.1 fatalities per 100,000 FTEs. In 2020, Alaskan workers had the 4^th^ highest rate of injuries (3.5 injuries per 100 FTEs) and the 3^rd^ highest rate of injuries with days away from work (1.8 injuries per 100 FTEs) [5].

The Alaska Occupational Safety and Health Section (AKOSH), within the Department of Labor and Workforce Development, is the state-based Occupational Safety and Health Administration (OSHA) Program. In the AKOSH Fiscal Year19-23 strategic plan the following industries were designated as high-hazard: (a) construction; (b) healthcare; (c) seafood processing; and (d) public sector [6]. Some of these industries were confirmed to have high injury rates as indicated by an analysis by the Alaska Department of Labor and Workforce Development, such as agriculture/forestry/fishing/hunting, transportation and material moving, and installation/maintenance/repair. Workers in the agriculture/forestry/fishing/hunting industry had the highest rate, with 11.0 injuries per 100 FTEs. When all injuries were categorized by Standard Occupational Classification (SOC), workers in the production (579.9 per 10,000 FTEs), transportation and material moving (305.4 per 10,000 FTEs), and installation/maintenance/repair (221.5 per 10,000 FTEs) occupations had the highest rates [7].

Recent studies on occupational injuries in Alaska have described injury risks among all workers, as well as those specifically in aviation, logging, commercial fishing, and seafood processing [4, 8–18]. Although workplace safety among young workers has captured national attention, none of the published studies to date have described injuries to young workers in Alaska beyond injury patterns among children (< 18 years of age) in the commercial fishing industry [18]. This is despite evidence of elevated risk of injury among Alaska’s young workers. In 2020, Bureau of Labor Statistics data showed that Alaska workers aged 16–19 and 20–24 years had nonfatal injury rates of 152.3 and 216.7 per 10,000 FTEs respectively [5]. The average injury rate for workers aged 25–54 years in Alaska was 173.1 per 10,000 FTEs in 2020. There is a clear need to better understand the injuries and hazards faced by young workers in the state. To accurately describe young worker injuries, it is crucial to combine multiple sources of injury data to provide the most complete description possible. The purpose of the present study was to use multiple data sources to describe the burden and characteristics of fatal and nonfatal traumatic occupational injuries among workers aged 24 years or younger in Alaska. In addition, we explored injuries to young workers in commercial fishing and seafood processing occupations in greater detail because these two industries have consistently been the most hazardous occupations in Alaska.

## Methods

### Case definition

This study included fatal and nonfatal occupational injuries among all young workers in Alaska during 2014–2018. To be included in this study, cases needed to be traumatic, work-related injuries sustained by workers aged 24 years or younger in Alaska or Alaskan waters.

### Data sources

#### Alaska workers’ compensation

In Alaska, the Division of Workers’ Compensation (AKWC) is charged with administering the Alaska Workers’ Compensation Act, which requires employers or their insurance carriers to pay for injured or ill employees’ work-related medical, disability, and reemployment benefits. Employers must report to the Division an employee's death, injury, disease, or infection that arises out of and in the course of employment [19]. Employers in the Alaska Workers’ Compensation system must report an employee’s death, injury, disease, or infection arising out of and in the course of employment to the State Division of Workers’ Compensation [20]. Commercial fishermen, federal government employees, self-employed, and military personnel are not captured in the AKWC data.

#### Alaska fishermen’s fund

Unlike most workers, fishermen are not covered by traditional workers’ compensation. Established in 1951, the Alaska Fishermen’s Fund (AFF) provides for the treatment and care of licensed commercial fishermen who have been injured while fishing on shore or off-shore in Alaska, including both residents and non-residents [21]. To be eligible for benefits, crewmembers with injury or illness directly connected to operations as a commercial fisherman must hold valid commercial fishing licenses or limited entry permits in Alaska or in Alaskan waters. Benefits are only awarded after consideration of other coverage, such as private health or vessel insurance. The AFF is a payer of last resort that is financed from revenue generated by the license and permit fees required for all commercial fishermen. Fishermen file AFF claims to cover transportation, medical care, prescriptions, and therapy costs.

#### Alaska trauma registry

Since 1991, the Alaska Trauma Registry (ATR) has collected data from all 24 of Alaska’s acute care hospitals for injured patients in Alaska, and the treatment they received. The purpose of the registry is to evaluate the quality of trauma patient care and to plan and evaluate injury prevention programs. Included in this dataset are patients who have injuries and who are admitted to an any of the 24 acute care hospitals, held for observation, transferred to another acute care facility, or declared dead in the emergency department, and for whom contact occurred within 30 days of the injury [22]. Data include injury information and patients’ treatments, outcomes, and demographics. Within the ATR dataset, there are variables that can be used to determine whether or not the injury occurred at work.

#### Alaska occupational injury surveillance system

The Alaska Occupational Injury Surveillance System (AOISS) is a comprehensive surveillance system designed and implemented by the National Institute for Occupational Safety and Health (NIOSH) to gather information on fatal occupational injuries. AOISS compiles risk factor information and permits quantitative epidemiologic analyses to be used for public health and prevention planning [23]. Fatal occupational injury information is collected from the State of Alaska, US Coast Guard investigations, National Transportation Safety Board reports, state and local police reports, and media. Cases in this dataset are fatal injuries sustained by workers in the state of Alaska while working.

### Data preparation and coding

A standard set of variables were created from all four sources of data. Some years of data for AKWC and all ATR were missing SOC codes. Therefore, the North American Industry Classification System (NAICS), job description, injury narrative, and/or position were used to code SOC. A combination of manual coding and the NIOSH Industry and Occupation Computerized Coding System (NIOCCS) [24] were used to fill in SOC codes whenever possible. Occupational Injury and Illness Classification System (OIICS) [25] codes were manually coded based on injury narratives or International Classification of Diseases-9 codes whenever OIICS codes were missing. Only injuries with an OIICS nature code of 1 at the 1-digit level were included in the final dataset. OIICS Nature codes with a first digit of 1 means the injury was a traumatic injury or disorder. Generally, a traumatic injury or disorder is the result of a single incident, event, or exposure over the course of a single shift [26].

For AKWC, the dataset included the date the patient started their time away from work, their return-to-work date, and a classification code describing the initial level of treatment required. To compare the severity of injuries across occupations, these variables were used to calculate the total number of days the individual was away from work. Categorical variables were then created to classify days away from work as: no time away, less than 7 days, between 7 and 30 days, and more than 30 days.

Standardized geographic region codes within the AKWC were applied to ATR, AOISS, and AFF cases to categorize where injuries occurred. The city or nearest city where the injury occurred were used to categorize non-AKWC cases into these economic regions: Anchorage/Matanuska Susitna (Mat-Su), Gulf Coast, Interior, Northern, Southwest, and Southeast. Seasons when the injury occurred were based on the month when the injury occurred (Winter: Dec-Feb, Spring: Mar-May, Summer: Jun-Aug, Fall: Sep-Nov). Based on the age of the individual at the time of the injury, three different age groups were used to categorize the workers: younger than 18, 18 to 19, and 20 to 24 years of age. These groups were selected based on previous research [1] that found that workers aged 18–19 years had the highest rates of injuries.

### Linking datasets

After the eligible cases were identified, duplicates were identified using a combination of sex, age, SOC, NAICS, city where the injury occurred, the date of injury, injury narratives, and/or OIICS variables. R studio (RStudio Team 2020. RStudio: Integrated Development Environment for R. RStudio, PBC, Boston, MA URL http://www.rstudio.com/) was used to merge the four datasets one at a time by sex, age, region, year, and month. Additionally, if the day when the injury occurred was present in both datasets, it was used to help identify possible duplicates. The resulting datasets were then manually filtered by day, month, and year to identify possible duplicates. When the temporal variables were a match, then workers’ age, sex, and region of the injury were used to identify possible duplicates. Lastly, the injury narrative and/or OIICS codes were used to conclusively identify duplicates. If variables were missing from one duplicate but present in the other, the information was copied over and then one duplicate was removed from the dataset. If the duplicate occurred between AKWC claims and any other data source, the AKWC claim was kept because it normally contained more information, while the other was removed. If the duplicate occurred between ATR case and AFF claim, the ATR case was kept while the AFF claim was removed.

### Analysis

Descriptive statistics, including frequency, percent distributions, and cross-tabulations between 2-digit SOC codes and 2-digit OIICS codes were calculated. We also calculated more detailed descriptive statistics on injury characteristics using more specific OIICS codes for commercial fishing and seafood processing cases in order to compare findings from our study to previous Alaska studies. R studio was used for data management, variable creation, duplicate search, and merging datasets. STATA 14.2 (StataCorp. 2015. Stata Statistical Software: Release 14. College Station, TX: StataCorp LP.) was used for all analyses and results. The NIOSH Institutional Review Board determined this study did not require a full review because its primary intent is surveillance for injury/illness control (IRB Number: 0900f3eb81c3f976).

## Results

### Characteristics of young worker injuries and fatalities

**Fig. 1:**
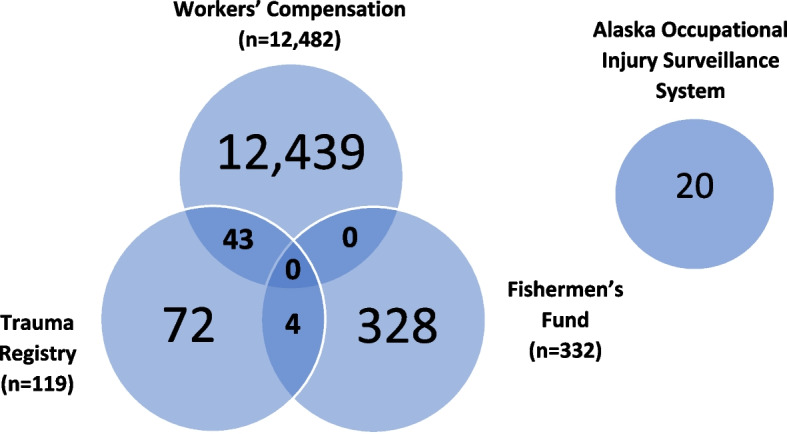
Nonfatal and fatal traumatic injuries among young workers in Alaska, 2014-2018, by dataset and linked (12,906 unique cases)

Overall, young workers aged 24 years or less represented 14% of all fatal and nonfatal occupational injuries across the four health data systems. Before excluding duplicates, young workers accounted for 19% of all AFF claims, 15% of all AKWC claims, 12% of all AOISS cases, and 5% of all occupational ATR cases. AKWC included 12,482 young worker claims, AOISS included 20 young worker fatalities, ATR included 119 young worker injuries, and AFF included 332 young worker claims before duplicates were removed. A total of 47 duplicates were identified between all the injury data systems (Fig. 1), resulting in a total of 12,906 unique cases. No duplicates were identified in more than two systems.

After removing duplicates, 20 work-related fatalities and 12,886 nonfatal injuries were included in this study. Over the 5-year period, approximately 2,577 nonfatal and four fatal injuries for young workers occurred every year in Alaska. The highest number of injuries occurred in 2014 (2,776) and appeared to decrease over time, with 2018 having the lowest number with 2,409 nonfatal injuries. The majority of injuries during 2014–2018 occurred in the Anchorage/Mat-Su (5,762, 45%) and Interior (2,130, 17%) regions (Table 1). Females experienced 38% (4,925) of the injuries while males experienced the remaining 62% (7,948). Among young workers less than 18 years of age, 47% were female while 53% were male. Injuries most often occurred during the summer (4,950, 38%) and the least during the winter (2,457, 19%). Workers 18–19 and 20–24 both experienced the highest percentage of injuries during the spring (18–19: 22%; 20–24: 22%) and summer (18–19: 40%; 20–24: 37%). In contrast, minors (< 18) were most frequently injured during the summer (50%) and winter (18%). Approximately 85% of injured workers were Alaskan residents.

**Table 1:** Characteristics of traumatic injuries and fatalities among young workers in Alaska, 2014–2018

		**Age Groups**			
		** < 18**	**18–19**	**20–24**	**Total**
**Total Injuries/Fatalities**	n	553	2,250	10,103	12,906
**Injury Type (** ***n*** ** = 12,906)**					
Non-Fatal Injury	n	552	2,246	10,088	12,886
	*col %*	*99%*	*99%*	*99%*	*99%*
Fatal Injury	n	1	4	15	20
	*col %*	< *1%*	< *1%*	< *1%*	< *1%*
**Alaska Region (** ***n*** ** = 12,776)**					
Anchorage/Mat-Su	n	287	1,009	4,466	5,762
	*col %*	*52%*	*45%*	*45%*	*45%*
Gulf Coast	n	71	252	1,105	1,428
	*col %*	*13%*	*11%*	*11%*	*11%*
Interior	n	82	369	1,679	2,130
	*col %*	*15%*	*17%*	*17%*	*17%*
Northern	n	10	101	485	596
	*col %*	*2%*	*4%*	*5%*	*5%*
Southeast	n	60	286	1,297	1,643
	*col %*	*11%*	*13%*	*13%*	*13%*
Southwest	n	39	206	972	1,217
	*col %*	*7%*	*9%*	*10%*	*9%*
**Sex (** ***n*** ** = 12,873)**					
Female	n	260	887	3,778	4,925
	*col %*	*47%*	*40%*	*38%*	*38%*
Male	n	292	1,360	6,296	7,948
	*col %*	*53%*	*60%*	*62%*	*62%*
**Season (** ***n*** ** = 12,906)**					
Spring	n	87	495	2,236	2,818
	*col %*	*16%*	*22%*	*22%*	*22%*
Summer	n	277	907	3,766	4,950
	*col %*	*50%*	*40%*	*37%*	*38%*
Fall	n	88	459	2,134	2,681
	*col %*	*16%*	*20%*	*21%*	*21%*
Winter	n	101	389	1,967	2,457
	*col %*	*18%*	*17%*	*20%*	*19%*
**Alaska Residency (** ***n*** ** = 12,906)**					
Non-Resident	n	61	363	1,471	1,895
	*col %*	*11%*	*16%*	*15%*	*15%*
Resident	n	492	1,887	8,632	11,011
	*col %*	*89%*	*84%*	*85%*	*85%*

### Injuries by occupation

Workers’ occupations using 2-digit SOC codes were identified for 10,250 (79%) of the young worker injuries and fatalities (Table 2). Production workers had the highest number of injuries during 2014–2018 (1,391, 14%). Workers in the food preparation and serving related occupations sustained 12% of injuries, followed by workers in the transportation and material moving occupations (11%). Sales occupations and construction/extraction occupations experienced 10% and 7% of the injuries, respectively. Of the 10,250 injuries to young workers, 4% occurred in workers aged 18 year or less, 18% in 18–19 and 78% in 20–24. The five occupations with the highest percentage of injuries to minors and more than 100 total injuries were food preparation/serving related (8%), farming/fishing/forestry (8%), sales and related (6%), transportation and material moving (5%), and business/financial services (5%).

**Table 2:** Traumatic injury counts by occupation^a^ and age groups

	**Production**	**Food Preparation, Serving Related**	**Transportation and Material Moving**	**Sales and Related**	**Construction and Extraction**	**Healthcare Support**	**Office and Administrative Support**	**Building and Grounds Cleaning and Maintenance**	**Personal Care and Service**	**Protective Service**	**Farming, Fishing, and Forestry**	**Installation, Maintenance, and Repair**
**Age Groups**	**N**	**N**	**N**	**N**	**N**	**N**	**N**	**N**	**N**	**N**	**N**	**N**
	***col %***	***col %***	***col %***	***col %***	***col %***	***col %***	***col %***	***col %***	***col %***	***col %***	***col %***	***col %***
< 18	27	99	62	62	14	9	23	20	20	6	37	11
	*2*	*8*	*5*	*6*	*2*	*1*	*4*	*4*	*4*	*1*	*8*	*3*
18–19	277	256	215	209	122	71	106	100	89	71	77	70
	*20*	*21*	*18*	*21*	*17*	*11*	*18*	*18*	*18*	*15*	*16*	*16*
20–24	1,087	870	889	712	598	546	462	438	400	396	358	353
	*78*	*71*	*76*	*72*	*81*	*87*	*78*	*78*	*79*	*84*	*76*	*81*
Total	1,391	1,225	1,166	983	734	626	591	558	509	473	473	434
	*100*	*100*	*100*	*100*	*100*	*100*	*100*	*100*	*100*	*100*	*100*	*100*

	**Educational Instruction and Library**	**Healthcare Practitioners and Technical**	**Business and Financial Operations**	**Management**	**Life, Physical, and Social Science**	**Arts, Design, Sports, Media, Entertainment**	**Community and Social Services**	**Military Specific Occupations**	**Architecture and Engineering**	**Computer and Mathematical**	**Legal**	**All Known Occupations**
**Age Groups**	**N**	**N**	**N**	**N**	**N**	**N**	**N**	**N**	**N**	**N**	**N**	**N**
	***col %***	***col %***	***col %***	***col %***	***col %***	***col %***	***col %***	***col %***	***col %***	***col %***	***col %***	***col %***
< 18	7	3	6	4	0	9	2	0	2	0	0	423
	*2*	*1*	*5*	*4*	*0*	*12*	*4*	*0*	*7*	*0*	*0*	*4*
18–19	41	21	18	14	15	18	3	4	4	2	0	1,803
	*13*	*8*	*16*	*14*	*18*	*24*	*5*	*13*	*13*	*25*	*0*	*18*
20–24	259	252	92	85	68	49	52	27	24	6	1	8,024
	*84*	*91*	*79*	*82*	*82*	*64*	*91*	*87*	*80*	*75*	*100*	*78*
Total	307	276	116	103	843	76	57	31	30	8	1	10,250
	*100*	*100*	*100*	*100*	*100*	*100*	*100*	*100*	*100*	*100*	*100*	*100*

^a^Standard Occupational Classification (SOC), 2-digit level

To identify the specific occupations in which young workers sustained the most injuries, we evaluated SOC codes at the 3-digit level. The top five occupations with the highest number of injuries were food processing (1,002, 10%), material moving (824, 8%), retail sales (812, 7%), construction trades (596, 6%), and cooks/food preparation (510, 5%) (Supplemental Table 1).

### Occupation and injury characteristics

Based on the nature (or type) of injury, 3,024 (29%) cases were classified as sprains/strains/tears, 1,955 (19%) were cuts/lacerations, 1,592 (15%) were bruises/contusions, 1,147 (11%) were non-specified injuries and disorders (for example: crushing injuries, soreness/pain/hurt, swelling/inflammation/irritation, numbness), and 614 (6%) were puncture wounds (except gunshot wounds). These five nature codes represented approximately 81% of all the injuries identified. Sprains/strains/tears were the leading type of injury for most of the occupations with over 100 total injuries, except for those in food preparation and serving, installation/maintenance/repair, educational instruction and library, and healthcare practitioners/technicians. The most common type of injury for food preparation and serving (404, 33%), installation/maintenance/repair (117, 27%), and educational instruction and library (82, 27%) was cuts/lacerations. Puncture wounds (except gunshot wounds) were the most common type of injury for healthcare practitioners/technicians (71, 26%). All other types of injuries by occupation are presented in Table 3.

**Table 3:** Traumatic injury counts by occupation^a^ and nature of Injury (2-digit level)

Types of traumatic injuries	Production	Food Preparation and Serving Related	Transportation, Material Moving	Sales and Related	Construction, Extraction	Healthcare Support	Office, Admin Support	Building, Grounds Cleaning. Maintenance	Personal Care and Service	Farming, Fishing, and Forestry	Protective Service	Installation, Maintenance, Repair
	**n**	**n**	**n**	**n**	**n**	**n**	**n**	**n**	**n**	**n**	**n**	**n**
	***col %***	***col %***	***col %***	***col %***	***col %***	***col %***	***col %***	***col %***	***col %***	***col %***	***col %***	***col %***
**Sprains, strains, tears**	429	218	412	321	204	172	217	194	167	151	150	111
	*31*	*18*	*35*	*33*	*28*	*27*	*37*	*35*	*33*	*32*	*32*	*26*
**Cuts, lacerations**	219	404	183	178	149	99	114	76	63	83	55	117
	*16*	*33*	*16*	*18*	*20*	*16*	*19*	*14*	*12*	*18*	*12*	*27*
**Bruises, contusions**	286	150	197	181	97	77	85	88	91	53	49	64
	*21*	*12*	*17*	*18*	*13*	*12*	*14*	*16*	*18*	*11*	*10*	*15*
**Nonspecified injuries and disorders**	160	116	133	124	89	55	71	68	39	45	85	47
	*11*	*9*	*11*	*13*	*12*	*9*	*12*	*12*	*8*	*10*	*18*	*11*
**Puncture wounds, except gunshot wounds**	59	22	48	34	33	151	22	37	53	20	15	20
	*4*	*2*	*4*	*3*	*4*	*24*	*4*	*7*	*10*	*4*	*3*	*5*
**Fractures**	66	34	47	18	44	7	19	20	24	43	6	15
	*5*	*3*	*4*	*2*	*6*	*1*	*3*	*4*	*5*	*9*	*1*	*3*
**Heat (thermal) burns**	33	136	18	32	14	5	12	3	6	3	6	10
	*2*	*11*	*1*	*3*	*2*	< *1*	*2*	< *1*	*1*	< *1*	*1*	*2*
**Abrasions, scratches**	13	30	31	16	29	22	16	12	16	8	8	16
	*1*	*2*	*3*	*2*	*4*	*3*	*3*	*2*	*3*	*2*	*2*	*4*
**Other traumatic injuries**	34	21	24	13	25	2	9	10	18	17	15	8
	*2*	*2*	*2*	*1*	*3*	< *1*	*1*	*2*	*3*	*4*	*3*	*2*
**Other poisoning, toxic, noxious, or allergenic effects**	25	23	13	15	12	4	2	9	4	8	49	6
	*2*	*2*	*1*	*1*	*2*	< *1*	< *1*	*2*	< *1*	*2*	*10*	*1*
**Concussions**	12	10	19	17	9	6	13	5	12	13	5	5
	< *1*	< *1*	*2*	*2*	*1*	*1*	*2*	< *1*	*2*	*3*	*1*	*1*
**Dermatitis and reactions affecting the skin–acute**	17	22	9	7	11	14	1	14	4	3	8	2
	*1*	*2*	< *1*	< *1*	*1*	*2*	< *1*	*2*	< *1*	< *1*	*2*	< *1*
**Chemical burns and corrosions**	5	16	13	14	6	7	6	13	4	7	12	5
	< *1*	*1*	*1*	*1*	< *1*	*1*	*1*	*2*	< *1*	*1*	*2*	*1*
**Dislocations**	15	10	11	9	4	3	2	5	5	8	7	4
	*1*	< *1*	< *1*	< *1*	< *1*	< *1*	< *1*	< *1*	*1*	*2*	*1*	< *1*
**Traumatic hernias**	11	9	5	2	5	1	0	2	1	3	2	1
	< *1*	< *1*	< *1*	< *1*	< *1*	< *1*	*0*	< *1*	< *1*	< *1*	< *1*	< *1*
**Amputations, avulsions, enucleations**	7	4	3	2	3	1	2	2	2	7	1	3
	< *1*	< *1*	< *1*	< *1*	< *1*	< *1*	< *1*	< *1*	< *1*	*1*	< *1*	< *1*
**Total**	1,391	1,225	1,166	983	734	626	591	558	509	472	473	434
	*100*	*100*	*100*	*100*	*100*	*100*	*100*	*100*	*100*	*100*	*100*	*100*

Types of traumatic injuries	Educational Instruction and Library	Healthcare Practitioners, Technical	Business, Financial Operations	Management	Life, Physical, Social Science	Arts, Design, Entertain, Sports, Media	Community and Social Services	Military Specific Occupations	Architecture and Engineering	Computer and Mathematical	Legal	Total with known occupation
	**n**	**n**	**n**	**n**	**n**	**n**	**n**	**n**	**n**	**n**	**n**	**n**
	***col %***	***col %***	***col %***	***col %***	***col %***	***col %***	***col %***	***col %***	***col %***	***col %***	***col*** ***%***	***col*** ***%***
**Sprains, strains, tears**	64	63	38	37	27	20	21	1	5	2	0	3,024
	*21*	*23*	*33*	*36*	*32*	*26*	*37*	*3*	*17*	*25*	*0*	*29*
**Cuts, lacerations**	82	43	15	20	14	19	2	6	10	3	1	1,955
	*27*	*16*	*13*	*19*	*17*	*25*	*3*	*19*	*33*	*37*	*100*	*19*
**Bruises, contusions**	70	21	24	17	12	12	11	1	6	0	0	1,592
	*23*	*8*	*21*	*16*	*14*	*16*	*19*	*3*	*20*	*0*	*0*	*15*
**Nonspecified injuries and disorders**	35	38	14	9	3	2	7	0	4	3	0	1,147
	*11*	*14*	*12*	*9*	*4*	*3*	*12*	*0*	*13*	*37*	*0*	*11*
**Puncture wounds, except gunshot wounds**	12	71	7	5	1	1	3	0	0	0	0	614
	*4*	*26*	*6*	*5*	*1*	*1*	*5*	*0*	*0*	*0*	*0*	*6*
**Fractures**	8	5	1	3	1	2	2	10	0	0	0	375
	*3*	*2*	*1*	*3*	*1*	*3*	*3*	*32*	*0*	*0*	*0*	*4*
**Heat (thermal) burns**	0	2	5	5	3	11	1	2	0	0	0	307
	*0*	< *1*	*4*	*5*	*4*	*14*	*2*	*6*	*0*	*0*	*0*	*3*
**Abrasions, scratches**	14	6	1	1	1	0	2	2	1	0	0	245
	*5*	*2*	< *1*	*1*	*1*	*0*	*3*	*6*	*3*	*0*	*0*	*2*
**Other traumatic injuries**	4	6	3	1	5	1	3	8	1	0	0	228
	*1*	*2*	*3*	*1*	*6*	*1*	*5*	*26*	*3*	*0*	*0*	*2*
**Other poisoning, toxic, noxious, or allergenic effects**	0	7	2	2	6	3	2	0	1	0	0	193
	*0*	*2*	*2*	*2*	*7*	*4*	*3*	*0*	*3*	*0*	*0*	*2*
**Concussions**	10	3	1	1	1	3	2	0	0	0	0	147
	*3*	*1*	< *1*	*1*	*1*	*4*	*3*	*0*	*0*	*0*	*0*	*1*
**Dermatitis and reactions affecting the skin–acute**	0	4	2	0	3	0	0	0	1	0	0	122
	*0*	*1*	*2*	*0*	*4*	*0*	*0*	*0*	*3*	*0*	*0*	*1*
**Chemical burns and corrosions**	1	4	0	1	4	2	0	0	0	0	0	120
	< *1*	*1*	*0*	*1*	*5*	*3*	*0*	*0*	*0*	*0*	*0*	*1*
**Dislocations**	4	2	1	0	0	0	0	0	1	0	0	91
	*1*	< *1*	< *1*	*0*	*0*	*0*	*0*	*0*	*3*	*0*	*0*	< *1*
**Traumatic hernias**	0	0	1	0	2	0	0	0	0	0	0	45
	*0*	*0*	< *1*	*0*	*2*	*0*	*0*	*0*	*0*	*0*	*0*	< *1*
**Amputations, avulsions, enucleations**	3	1	1	1	0	0	1	1	0	0	0	45
	*1*	< *1*	< *1*	*1*	*0*	*0*	*2*	*3*	*0*	*0*	*0*	< *1*
**Total**	307	276	116	103	83	76	57	31	30	8	1	10,250
	*100*	*100*	*100*	*100*	*100*	*100*	*100*	*100*	*100*	*100*	*100*	*100*

^a^ Standard Occupational Classification (SOC), 2-digit level

The cause of injuries (event/exposure in OIICS) was coded for 9,828 (76%) injuries. The leading causes of injuries were struck by object or equipment (2,027, 21%), overexertion involving outside sources (1,385, 14%), struck against object or equipment (905, 9%), falls on same level (728, 7%), and exposure to other harmful substances (636, 6%). Struck by object or equipment was the leading cause of injuries for young worker occupations with more than 100 total cases except those in the healthcare support, personal care and service, farming/fishing/forestry, healthcare practitioners/technical, and business and financial operations. Exposure to other harmful substances was the most common cause of injuries for healthcare support workers (154, 26%), protective services (57, 14%), and healthcare practitioners/technical (61, 24%). Falls on same level were the leading cause of injuries for personal care and service workers (67, 14%) while water vehicle incidents were the most common for farming/fishing/forestry workers (325, 72%). For business and financial operations, overexertion involving outside sources (26, 23%) was the most common cause of injuries. All other causes of injuries by occupation are presented in Table 4.

**Table 4:** Traumatic Injury counts by occupation^a^ and event/exposure (2-digit level)

Causes of traumatic injuries	Production	Food Preparation and Serving Related	Transportation, Material Moving	Sales and Related	Construction, Extraction	Healthcare Support	Office and Administrative Support	Building, Grounds Cleaning, Maintenance	Personal Care and Service	Farming, Fishing, and Forestry	Installation, Maintenance, and Repair	Protective Service
	**n**	**n**	**n**	**n**	**n**	**n**	**n**	**n**	**n**	**n**	**n**	**n**
	***col %***	***col %***	***col %***	***col %***	***col %***	***col %***	***col %***	***col %***	***col %***	***col %***	***col %***	***col %***
**Struck by object or equipment**	320	352	238	211	205	88	112	99	56	23	103	35
	*24*	*29*	*21*	*22*	*29*	*15*	*19*	*19*	*11*	*5*	*25*	*9*
**Overexertion involving outside sources**	265	119	188	115	127	110	95	80	62	14	59	47
	*20*	*10*	*17*	*12*	*18*	*18*	*17*	*15*	*13*	*3*	*14*	*11*
**Struck against object or equipment**	191	119	92	110	69	27	56	60	33	11	58	19
	*14*	*10*	*8*	*12*	*10*	*4*	*10*	*11*	*7*	*2*	*14*	*5*
**Falls on same level**	84	83	91	64	44	19	63	37	67	7	24	23
	*6*	*7*	*8*	*7*	*6*	*3*	*11*	*7*	*14*	*2*	*6*	*6*
**Exposure to other harmful substances**	45	63	38	37	27	154	15	50	19	27	11	57
	*3*	*5*	*3*	*4*	*4*	*26*	*3*	*9*	*4*	*6*	*3*	*14*
**Other exertions or bodily reactions**	43	38	76	115	22	16	40	30	25	7	17	20
	*3*	*3*	*7*	*12*	*3*	*3*	*7*	*6*	*5*	*2*	*4*	*5*
**Contact with objects and equipment, unspecified**	52	83	57	60	47	14	28	18	14	11	20	19
	*4*	*7*	*5*	*6*	*7*	*2*	*5*	*3*	*3*	*2*	*5*	*5*
**Intentional Injury by person**	9	25	66	33	1	54	31	21	39	0	6	36
	< *1*	*2*	*6*	*3*	< *1*	*9*	*5*	*4*	*8*	*0*	*1*	*9*
**Water vehicle incident**	21	2	7	2	1	0	1	0	9	325	2	1
	*2*	< *1*	< *1*	< *1*	< *1*	*0*	< *1*	*0*	*2*	*72*	< *1*	< *1*
**Caught in or compressed by equipment or objects**	106	46	52	26	30	5	19	12	1	3	20	9
	*8*	*4*	*5*	*3*	*4*	< *1*	*3*	*2*	< *1*	< *1*	*5*	*2*
**Falls to lower level**	39	30	35	23	37	27	11	36	25	3	17	27
	*3*	*2*	*3*	*2*	*5*	*4*	*2*	*7*	*5*	< *1*	*4*	*7*
**Exposure to temperature extremes**	41	137	19	33	15	6	11	4	6	1	7	7
	*3*	*11*	*2*	*3*	*2*	*1*	*2*	< *1*	*1*	< *1*	*2*	*2*
**Animal or insect**	34	3	19	20	4	13	10	5	49	3	19	4
	*2*	< *1*	*2*	*2*	< *1*	*2*	*2*	*1*	*10*	< *1*	*5*	*1*
**Slip or trip without fall**	22	15	34	36	10	7	22	14	10	4	5	17
	*2*	*1*	*3*	*4*	*1*	*1*	*4*	*3*	*2*	< *1*	*1*	*4*
**Injury by person–unintentional or unknown**	5	7	18	11	3	25	15	6	18	1	4	27
	< *1*	< *1*	*2*	*1*	< *1*	*4*	*3*	*1*	*4*	< *1*	*1*	*7*
**Roadway incidents involving motorized land vehicles**	5	13	32	6	4	1	10	13	27	1	4	7
	< *1*	*1*	*3*	< *1*	< *1*	< *1*	*2*	*2*	*5*	< *1*	*1*	*2*
**Rubbed or abraded by friction or pressure**	21	27	14	9	30	5	7	2	2	5	14	8
	*2*	*2*	*1*	*1*	*4*	< *1*	*1*	< *1*	< *1*	*1*	*3*	*2*
**Other Transportation**	9	2	21	8	7	1	11	23	14	3	3	4
	< *1*	< *1*	*2*	< *1*	*1*	< *1*	*2*	*4*	*3*	< *1*	< *1*	*1*
**Other fall, slip, trip**	15	8	12	7	2	4	9	5	9	0	5	7
	*1*	< *1*	*1*	< *1*	< *1*	< *1*	*2*	*1*	*2*	*0*	*1*	*2*
**Other overexertion and bodily reaction**	14	4	11	8	1	7	5	6	1	2	2	11
	*1*	< *1*	*1*	< *1*	< *1*	*1*	< *1*	*1*	< *1*	< *1*	< *1*	*3*
**Fires or Explosions**	3	11	3	0	2	0	0	0	0	0	10	19
	< *1*	< *1*	< *1*	*0*	< *1*	*0*	*0*	*0*	*0*	*0*	*2*	*5*
**Other contact with objects and equipment**	0	3	2	2	3	17	2	2	3	1	2	1
	*0*	< *1*	< *1*	< *1*	< *1*	*3*	< *1*	< *1*	< *1*	< *1*	< *1*	< *1*
**Other exposures to harmful substances or environment**	7	4	6	5	13	0	1	3	1	0	3	2
	< *1*	< *1*	< *1*	< *1*	*2*	*0*	< *1*	< *1*	< *1*	*0*	< *1*	< *1*
**Total**	1,351	1,194	1,131	941	704	600	574	526	490	451	415	408
	*100*	*100*	*100*	*100*	*100*	*100*	*100*	*100*	*100*	*100*	*100*	*100*

Causes of traumatic injuries	Educational Instruction and Library	Healthcare Practitioners, Technical	Business and Financial Operations	Management	Life, Physical, and Social Science	Arts, Design, Entertain, Sports, Media	Community and Social Services	Military Specific Occupations	Architecture and Engineering	Computer and Mathematical	Legal	Total with known occupation
	**n**	**n**	**n**	**n**	**n**	**n**	**n**	**n**	**n**	**n**	**n**	**n**
	***col %***	***col %***	***col %***	***col %***	***col %***	***col %***	***col %***	***col %***	***col %***	***col %***	***col %***	***col %***
**Struck by object or equipment**	73	33	22	16	14	15	2	2	7	1	0	2,027
	*24*	*13*	*19*	*16*	*18*	*20*	*4*	*6*	*25*	*14*	*0*	*21*
**Overexertion involving outside sources**	16	23	26	12	13	9	2	0	2	1	0	1,385
	*5*	*9*	*23*	*12*	*16*	*12*	*4*	*0*	*7*	*14*	*0*	*14*
**Struck against object or equipment**	11	11	8	10	4	7	5	0	2	2	0	905
	*4*	*4*	*7*	*10*	*5*	*10*	*9*	*0*	*7*	*29*	*0*	*9*
**Falls on same level**	53	18	10	11	6	9	9	1	3	2	0	728
	*18*	*7*	*9*	*11*	*8*	*12*	*16*	*3*	*11*	*29*	*0*	*7*
**Exposure to other harmful substances**	1	61	8	4	9	5	2	1	2	0	0	636
	< *1*	*24*	*7*	*4*	*11*	*7*	*4*	*3*	*7*	*0*	*0*	*6*
**Other exertions or bodily reactions**	11	9	6	14	6	2	0	0	0	0	0	497
	*4*	*3*	*5*	*14*	*8*	*3*	*0*	*0*	*0*	*0*	*0*	*5*
**Contact with objects and equipment, unspecified**	16	10	10	2	2	2	1	0	0	1	1	468
	*5*	*4*	*9*	*2*	*2*	*3*	*2*	*0*	*0*	*14*	*100*	*5*
**Intentional Injury by person**	74	23	0	3	3	2	7	6	0	0	0	439
	*25*	*9*	*0*	*3*	*4*	*3*	*13*	*19*	*0*	*0*	*0*	*4*
**Water vehicle incident**	1	0	0	1	1	0	4	0	0	0	0	378
	< *1*	*0*	*0*	*1*	*1*	*0*	*7*	*0*	*0*	*0*	*0*	*4*
**Caught in or compressed by equipment or objects**	1	5	3	5	1	3	3	2	4	0	0	356
	< *1*	*2*	*3*	*5*	*1*	*4*	*5*	*6*	*14*	*0*	*0*	*4*
**Falls to lower level**	8	6	4	5	2	1	0	2	3	0	0	341
	*3*	*2*	*3*	*5*	*2*	*1*	*0*	*6*	*11*	*0*	*0*	*3*
**Exposure to temperature extremes**	0	2	6	5	3	11	1	0	0	0	0	315
	*0*	< *1*	*5*	*5*	*4*	*15*	*2*	*0*	*0*	*0*	*0*	*3*
**Animal or insect**	3	18	3	2	6	0	1	1	0	0	0	217
	*1*	*7*	*3*	*2*	*8*	*0*	*2*	*3*	*0*	*0*	*0*	*2*
**Slip or trip without fall**	2	2	3	2	0	1	3	0	0	0	0	209
	< *1*	< *1*	*3*	*2*	*0*	*1*	*5*	*0*	*0*	*0*	*0*	*2*
**Injury by person–unintentional or unknown**	22	14	3	3	0	3	5	3	0	0	0	193
	*7*	*5*	*3*	*3*	*0*	*4*	*9*	*10*	*0*	*0*	*0*	*2*
**Roadway incidents involving motorized land vehicles**	7	2	1	3	1	2	4	2	2	0	0	147
	*2*	< *1*	< *1*	*3*	*1*	*3*	*7*	*6*	*7*	*0*	*0*	*1*
**Rubbed or abraded by friction or pressure**	0	1	0	0	1	1	0	0	0	0	0	147
	*0*	< *1*	*0*	*0*	*1*	*1*	*0*	*0*	*0*	*0*	*0*	*1*
**Other Transportation**	0	0	0	0	1	0	1	8	0	0	0	116
	*0*	*0*	*0*	*0*	*1*	*0*	*2*	*26*	*0*	*0*	*0*	*1*
**Other fall, slip, trip**	1	2	0	1	0	0	2	0	1	0	0	90
	< *1*	< *1*	*0*	*1*	*0*	*0*	*4*	*0*	*4*	*0*	*0*	< *1*
**Other overexertion and bodily reaction**	0	0	0	1	2	0	2	0	2	0	0	79
	*0*	*0*	*0*	*1*	*2*	*0*	*4*	*0*	*7*	*0*	*0*	< *1*
**Fires or Explosions**	0	1	0	1	4	0	0	3	0	0	0	57
	*0*	< *1*	*0*	*1*	*5*	*0*	*0*	*10*	*0*	*0*	*0*	< *1*
**Other contact with objects and equipment**	0	14	0	0	0	0	0	0	0	0	0	52
	*0*	*5*	*0*	*0*	*0*	*0*	*0*	*0*	*0*	*0*	*0*	< *1*
**Other exposures to harmful substances or environment**	0	0	0	0	0	0	1	0	0	0	0	46
	*0*	*0*	*0*	*0*	*0*	*0*	*2*	*0*	*0*	*0*	*0*	< *1*
**Total**	300	255	113	101	79	73	55	31	28	7	1	9,828
	*100*	*100*	*100*	*100*	*100*	*100*	*100*	*100*	*100*	*100*	*100*	*100*

^a^Standard Occupational Classification (SOC), 2-digit level

Using the full SOC and NAICS codes, we were able to identify 342 (3%) commercial fishing and 829 (6%) seafood processing injuries for young workers in Alaska. For commercial fishing workers, approximately 27% (93) of the injuries were to the hand(s), wrists, and fingers while lumbar injuries made up 13% (45) (Supplemental Table 2). The top three types of injuries to commercial fisherman were cuts/lacerations (65, 19%), strains (48, 14%), and fractures (43, 13%) (Supplemental Table 3). Similar to commercial fishing workers, seafood processing workers experienced 26% (216) of their injuries to the hand(s) while injuries to the back (88) made up another 11% (Supplemental Table 4). The top three types of injuries to seafood processing workers were sprains/strains/tears (317, 38%), bruises/contusions (179, 22%), and cuts/lacerations (93, 11%) (Supplemental Table 5).

### Occupation and injury severity for workers’ compensation claims

For the workers’ compensation claims, the dates for their last day of work, return to work, and claim codes (a code used to determine if the claim required time away) were used to categorize the number of days away from work due to the injury. Only 9,828 (79%) AKWC claims contained SOC codes and valid days away from work calculations. For the majority of the claims (82%), no time away from work was required. The occupations with the highest percentage of injuries that required at least 1 day away from work were personal care and service (24%), production (23%), construction and extraction (22%), transportation and material moving (21%), and installation/maintenance/repair (21%). The occupations with the largest percentage of injuries that required more than 30 days away from work were production (12%), transportation and material moving (9%), construction and extraction (12%), and personal care and service (11%), and arts/design/entertainment/sports/media (9%). The severity categories for all other occupations can be found in Table 5.

**Table 5:** Traumatic injury counts by occupation^a^ and severity^b^

	**Production**	**Food Preparation and Serving Related**	**Transportation and Material Moving**	**Sales and Related**	**Construction and Extraction**	**Healthcare Support**	**Office and Administrative Support**	**Building and Grounds Cleaning and Maintenance**	**Personal Care and Service**	**Protective Service**	**Installation, Maintenance, and Repair**	**Educational Instruction and Library**
**Severity**	**n**	**n**	**n**	**n**	**n**	**n**	**n**	**n**	**n**	**n**	**n**	**n**
	***col %***	***col %***	***col %***	***col %***	***col %***	***col %***	***col %***	***col %***	***col %***	***col %***	***col %***	***col %***
No time away	1,052	1,016	917	829	559	556	487	455	385	413	343	268
	*77*	*83*	*79*	*84*	*78*	*89*	*82*	*82*	*76*	*87*	*79*	*87*
Less than 7 days	89	75	80	43	37	25	44	28	29	17	29	18
	*6*	*6*	*7*	*4*	*5*	*4*	*7*	*5*	*6*	*4*	*7*	*6*
Between 7 and 30 days	71	60	60	41	40	23	24	37	33	23	31	11
	*5*	*5*	*5*	*4*	*6*	*4*	*4*	*7*	*6*	*5*	*7*	*4*
More than 30 days	160	74	99	69	84	22	35	38	57	20	29	10
	*12*	*6*	*9*	*7*	*12*	*3*	*6*	*7*	*11*	*4*	*7*	*3*
Total	1,372	1,225	1,156	982	720	626	590	558	504	473	432	307
	*100*	*100*	*100*	*100*	*100*	*100*	*100*	*100*	*100*	*100*	*100*	*100*

	**Healthcare Practitioners and Technical**	**Farming, Fishing, and Forestry**	**Business and Financial Operations**	**Management**	**Life, Physical, and Social Science**	**Arts, Design, Entertainment, Sports, and Media**	**Community and Social Services**	**Architecture and Engineering**	**Computer and Mathematical**	**Legal**	**Total**
**Severity**	**n**	**n**	**n**	**n**	**n**	**n**	**n**	**n**	**n**	**n**	**n**
	***col %***	***col %***	***col %***	***col %***	***col %***	***col %***	***col %***	***col %***	***col %***	***col %***	***col %***
No time away	242	115	94	88	70	65	49	26	8	1	8,038
	*88*	*86*	*81*	*85*	*84*	*87*	*86*	*87*	*100*	*100*	*82*
Less than 7 days	8	7	6	5	5	0	5	0	0	0	550
	*3*	*5*	*5*	*5*	*6*	*0*	*9*	*0*	*0*	*0*	*6*
Between 7 and 30 days	8	4	8	3	3	3	1	3	0	0	487
	*3*	*3*	*7*	*3*	*4*	*4*	*2*	*10*	*0*	*0*	*5*
More than 30 days	18	8	8	7	5	7	2	1	0	0	753
	*7*	*6*	*7*	*7*	*6*	*9*	*3*	*3*	*0*	*0*	*8*
Total	276	134	116	103	83	75	57	30	8	1	9,828
	*100*	*100*	*100*	*100*	*100*	*100*	*100*	*100*	*100*	*100*	*100*

^a^Standard Occupational Classification (SOC), 2-digit level

^b^Severity defined as the number of days away from work. Only available for AKWC claims

### Characteristics of fatal injuries

During 2014–2018, 20 fatalities among young workers were captured by the AOISS dataset. Most fatalities (17, 85%) occurred in workers aged 20–24. The remainder of the fatalities (3, 15%) occurred in workers between the ages of 18–19; no deaths occurred among minors. Of the 20 fatalities, 14 (70%) of the fatalities were residents of Alaska while the remaining 6 (30%) were not residents. The majority of the fatalities (12, 60%) occurred during the months of June and July while four (20%) occurred during September and October. The remaining four (20%) fatalities occurred between February and April.

Based on the SOC codes, 6 (30%) fatalities occurred in transportation and material moving, 5 (25%) occurred in military, 4 (20%) in the fishers and related fishing, 3 (15%) in construction and extraction, and 2 (10%) in personal care and service occupations. Blunt force trauma was responsible for 9 (45%) fatalities and drownings or presumed drownings were the cause of death for 5 (25%) young workers. Suicide by handgun was the cause of death for 3 (15%) fatalities and an unintentional gunshot wound to the head was attributed to one (5%) fatality. The cause of death for the last 2 (10%) young workers were crushing and traumatic asphyxia.

## Discussion

### Importance of linking multiple data systems

This study characterized fatal and nonfatal injuries among young workers aged 24 or younger in the state of Alaska by linking four separate health data systems (AKWC, AOISS, ATR, AFF). Linking these systems and identifying duplicate injuries allowed for a comprehensive evaluation of traumatic injuries in this group of Alaskan workers.

During the 5-year study period of 2014–2018, there were 12,886 nonfatal traumatic injuries and 20 fatalities reported. Four duplicates were identified between ATR cases and AFF claims, zero between AFF claims and AKWC claims, and 43 duplicates between ATR cases and AKWC claims. No duplicates were identified between fatal AOISS cases and any of the other occupational health surveillance systems. The lack of duplicates across these datasets highlights the need to incorporate information across multiple data sources. For example, over 99% of the commercial fishing and offshore seafood processing injuries from AFF were not present in AKWC or ATR. Without incorporating all four of these datasets for this study, we would have missed a significant proportion (~ 28%) of young worker injuries in the commercial fishing and offshore seafood processing industries.

### Comparison to injuries among all US young workers

A Morbidity and Mortality Week Report (MMWR) published in 2020 examined nonfatal occupational injuries involving young workers across the US. The authors found that while rates of injuries treated in hospital emergency departments generally decreased from 2012 to 2018, workers aged 15–24 years experienced higher rates of injury compared to those aged 25–44 years. For young workers, the occupations with the highest percentage of injuries with at least one day away from work were accommodation and food service (45.0%), transportation and material moving (14.7%), sales and related (8.7%), office and administrative support (5.7%), and production (8.5%) [1]. One of the biggest differences between Alaska and US young worker injuries is that the percentage of injuries requiring at least one day away was significantly higher for many occupations in Alaska. The only occupation that had a higher percentage of injuries requiring at least one day away in the MMWR report, compared to the top five Alaskan occupations, was accommodation and food service (45%). We found that occupations with the highest percentage of injuries that required at least one day away for Alaska workers were personal care and service (24%), production (23%), construction and extraction (22%), transportation and material moving (21%), and installation/maintenance/repair (21%). These similarities highlight that young workers in Alaska are experiencing a large number of injuries in many of the same occupations as young workers in the rest of the US. The key difference is the specific types of occupations in which young workers in Alaska are sustaining injuries. For example, Alaska’s young workers in construction and extraction occupations had the third highest percentage of injuries (22%) while construction workers were only the sixth highest percentage (8%) for the rest of the country. When the most common types of injury (nature) and event or exposure (source) were compared between the MMWR article and this study, we found that young workers in Alaska and the United States had very similar injury characteristics.

### Comparison to injuries among all ages of workers in Alaska

The demographic profile of young workers in this study mirrored the findings from a previously published study of injuries among all workers in Alaska using workers’ compensation claims during 2014–2015 [4]. Characteristics such as Alaska residency, sex, and geographic region had similar distributions. However, the most prevalent hazards and resulting injuries diverged between these populations. The top five occupations of injured young workers were food processing, material moving, retail sales, construction, and cooking; while for injured workers of all ages, the top five occupations were construction, food processing, material moving, building cleaning, and health practitioners [4].

Injury characteristics among young workers were somewhat different than among all workers in Alaska. Although sprains, strains, and tears were the leading injury among both populations, they represented 45.0% of injuries among all workers but 33.4% among young workers. Lacerations were more prevalent among young workers than among all workers. These findings are likely a reflection of the different hazards faced by young workers in their most prevalent occupations. For instance, the leading hazard injuring all workers was overexertion with an object. Among young workers, the leading hazard was struck by an object. These differences highlight the importance of our findings for targeting specific safety solutions to young workers.

### Commercial fishing and seafood processing workers

Alaska’s economically vital and hazardous seafood industry includes the interconnected and overlapping commercial fishing and seafood processing industries, which initiate the supply of seafood to consumers around the world. Commercial fishing occurs offshore, with workers located onboard various fleets of vessels that use tailored gear to harvest and/or process different species within a geographic region. Seafood processing occurs in factories, with workers located both offshore in vessels and onshore in buildings. Young workers in these seafood industries experienced very similar injury patterns compared to workers of all ages.

For Alaska’s commercial fishing industry, a previous study of nonfatal injury/illness during 2012–2016 linked data from ATR, AFF, and US Coast Guard reports [14]. This included incidents onboard vessels that harvested and/or processed seafood. During the 5-year study period, 3,014 unique injury/illness cases were identified among fishermen aged 11 to 82 years. By nature of injury/illness, most cases were traumatic injuries (2,779, 92.5%) and the most common injuries included: sprains/strains/tears (819); cuts (452); and fractures (343). The event or exposure resulting in injury/illness was most often contact with objects/equipment (1,101, 41%); overexertion and bodily reaction (738, 27%); and slips/trips/falls (541, 20%). The current study among young workers identified the same patterns of injury types. Unfortunately, it is not possible to directly compare the event/exposure resulting in injury between all workers and young workers, given differences in coding rules between the studies, with the previous study using rules specific to NIOSH’s Commercial Fishing Incident Database and this study using traditional OIICS coding rules.

For Alaska’s onshore seafood processing industry, a previous study of nonfatal injury/illness during 2014–2015 analyzed workers’ compensation claims data [16]. During the 2-year study period, 2,889 claims were accepted for compensation, among workers aged 16 to 79 years old, with a median of 37 years. By nature of injury/illness, cases were most commonly: sprains, strains, tears (993, 36%); bruises (490, 18%); and lacerations, punctures, and amputations (349, 13%). These were most often caused by contact with objects and equipment (1020, 37%); overexertion and bodily reaction (933, 34%); and slips, trips, and falls (448, 16%). In comparison, the current study among young workers identified very similar types and causes of traumatic injuries.

### Recommendations for preventing injuries

Young workers are at high-risk for traumatic injury and have become a focus of occupational safety research and prevention in the United States [1, 27]. This study found that young workers are experiencing high numbers of traumatic injuries in occupations that are known to be hazardous to adult workers in Alaska and the rest of the US, such as construction and production. This highlights the importance of identifying the causes of these injuries through research and implementing interventions to lower the risks associated with working in specific industries. For example, we have seen significant progress in reducing the number of occupational injuries and fatalities in the US commercial fishing industry. Despite this progress, commercial fishing in Alaska is still one of the most hazardous occupations and it is not uncommon for workers under 25 years of age to be present on commercial fishing vessels. Researchers across the United States need to continue to focus on these high-hazard industries and occupations in their local communities because removing or reducing the hazards for all workers will have an impact on young workers’ health as well.

Employers, parents, educators, and young workers themselves all play a large role in ensuring that young workers are protected from harm in the workplace [27]. Employers have a responsibility to protect young workers by maintaining safe and healthy workplaces. This includes complying with safety, health, and child labor laws; closely supervising young workers and developing a good safety culture in which young workers can feel confident expressing concerns; and delivering job-specific safety training [3]. Parents can help ensure their child’s safety by actively participating in educating themselves about child labor laws, talking with their child about their work, and helping them understand their right to a safe workplace. Educators can also play a key role in keeping young workers safe by incorporating school-based work experience programs, including worker safety and health curriculums in their course, and providing students with resources on occupational safety and health. Lastly, the young workers themselves can play a key role by being proactive in learning about labor laws and requesting safety training for specific workplace hazards. They can also refuse to perform unsafe tasks or work in unsafe conditions.

### Resources for improving young worker safety

There are several freely available resources to help improve workplace safety for young workers. Talking Safety, which was developed and evaluated by NIOSH and its partners, is a free curriculum that helps teachers and community-based job placement staff educate young workers about the basics of job safety and health [24]. This includes the identification of workplace hazards, methods for addressing them, and their rights and responsibilities. An Alaska-specific curriculum has been developed and is readily available [28]. The #KeepTeenWorkersSafe media campaign was created to increase awareness and promote workplace safety and health resources for young workers, and help disperse information to young workers, parents, teachers, and employers [29]. The National Young Worker Safety Resource Center (YWSRC) is a collaborative project of University of California Berkeley’s Labor Occupational Health Program and the Education Development Center, Inc [30]. This resource center provides consultations and referrals for occupational health practitioners, training programs for teachers, follow up assistance, and media content about youth employment for small business organizations.

### Limitations and future directions

The findings from this study are subject to a few limitations. First, this study was unable to calculate injury rates due to the lack of high-quality workforce employment statistics by occupation or industry for young workers in Alaska. Some of the main limitations of using the United States or Alaska workforce employment estimates are that they do not include non-resident workers and the industry specific estimates are not broken down by age groups for specific NAICS or SOC codes. Although we were unable to quantify the risk of injuries for specific occupations or industries, our study describes the occupations with the largest number of traumatic injuries as well as the types, causes, and severity of injuries. Additionally, it is likely young worker injuries in Alaska are being underreported. A study of Canadian young workers found that over half of individuals who experienced a lost time injury reported the work-related injury to both their employers and a doctor while 27% failed to report the time lost injury to their employer or a doctor [31]. Although, we are confident that the duplicates identified in this study are true duplicates, it is possible that we missed some duplicates due to data being input incorrectly.

Future research could focus on young worker injuries and fatalities for high-risk groups of workers such as those in commercial fishing or seafood processing. Next, although this study used four different data systems, there are undoubtedly cases that were not reported or identified. It is expected that underreporting of work-related injuries occurs [31]. Factors related to underreporting, such as fear of reprisal, may be compounded in young and inexperienced workers. Finally, future research could also explore how young workers’ risk perception and acceptance of occupational injuries plays a role in the choice of high-risk occupations such as commercial fishing. Target campaigns for young workers should also be developed to help them understand the importance of and to encourage injury reporting.

## Conclusion

The results from this analysis highlight the need for additional outreach and intervention to prevent injuries to young workers in Alaska while also informing future research. Although young workers participate in a variety of different occupations in the US, a significant proportion of the injuries to young workers in Alaska occurred in previously identified high risk occupations. Commercial fishing, seafood production, construction and extraction, and personal care occupations had the highest percentage of injuries that required more than one day away from work. Future research should focus on determining the rates of injuries for young workers in Alaska by occupation and severity, as well as developing and evaluating interventions to prevent injuries.

## Supplementary Information

**Additional file 1.**

## Data Availability

The data collected during this study will not be available from the corresponding author upon reasonable request. The agreements between NIOSH and the State of Alaska Department of Labor and Workforce Development, Division of Workers’ Compensation does not include language about sharing any of the AFF or AKWC data with a third party. Richard Evoy can be contacted for data requests at qom1@cdc.gov.
