# Supplementary material for: Traumatic injuries among Alaska’s young workers: Linking cases from four data systems
Source: BMC Public Health. 2023 Jan 9;23:57. doi: 10.1186/s12889-022-14676-7 (PMC9830688; doi:10.1186/s12889-022-14676-7)
Supplement: Supplementary file 1 — Additional file 1. [file 12889_2022_14676_MOESM1_ESM.docx]

**Supplemental Table 1: Top 20 Occupations (Standard Occupational Classification* at 3-digit level) by number of cases***

| **SOC Title (3-digit level)** | **SOC (3-digit level)** | **Frequency** | **Percentage** |
| --- | --- | --- | --- |
| Food Processing Workers | 513 | 1,002 | 10% |
| Material Moving Workers | 537 | 824 | 8% |
| Retail Sales Workers | 412 | 812 | 8% |
| Construction Trades Workers | 472 | 596 | 6% |
| Cooks and Food Preparation Workers | 352 | 510 | 5% |
| Food and Beverage Serving Workers | 353 | 470 | 5% |
| Home Health and Personal Care Aides | 311 | 417 | 4% |
| Building Cleaning and Pest Control Workers | 372 | 406 | 4% |
| Fishing and Hunting Workers | 453 | 342 | 3% |
| Other Personal Care and Service Workers | 399 | 276 | 3% |
| Material Recording, Scheduling, Dispatching, and Distributing Workers | 435 | 261 | 3% |
| Other Production Occupations | 519 | 219 | 2% |
| Vehicle and Mobile Equipment Mechanics, Installers, and Repairers | 493 | 206 | 2% |
| Other Healthcare Support Occupations | 319 | 204 | 2% |
| Firefighting and Prevention Workers | 332 | 201 | 2% |
| Information and Record Clerks | 434 | 197 | 2% |
| Other Food Preparation and Serving Related Workers | 359 | 182 | 2% |
| Other Installation, Maintenance, and Repair Occupations | 499 | 181 | 2% |
| Motor Vehicle Operators | 533 | 178 | 2% |
| Health Technologists and Technicians | 292 | 173 | 2% |
| **All occupations** | **All** | **10,250** | **100.0%** |

* 2,656 cases were missing SOC codes

**Supplemental Table 2: Top Occupational Injury and Illness Classification System (OIICS) Body Part for Commercial Fisherman**

| **Body Part Title** | **Body Part** | **Freq.** | **Percent** |
| --- | --- | --- | --- |
| Lumbar region | 322 | 45 | 13% |
| Finger(s), fingernail(s), unspecified | 4420 | 38 | 11% |
| Hand(s), unspecified | 440 | 24 | 7% |
| Finger(s), fingernail(s), n.e.c. | 4429 | 19 | 6% |
| Eye(s) | 132 | 14 | 4% |
| Knee(s) | 512 | 14 | 4% |
| Wrist(s) | 43 | 12 | 3% |
| Brain | 111 | 12 | 3% |
| Ankle(s) | 52 | 11 | 3% |
| Lower leg(s) | 513 | 11 | 3% |
| Shoulder(s), including clavicle(s), scapula(e) | 41 | 10 | 3% |
| Back, including spine, spinal cord, unspecified | 320 | 8 | 2% |
| Thoracic region | 321 | 8 | 2% |
| Forearm(s) | 423 | 8 | 2% |
| Hand(s), except finger(s) | 441 | 8 | 2% |
| Body Systems | 6 | 7 | 2% |
| Head, unspecified | 10 | 7 | 2% |
| Abdomen, except internal location of diseases or disorders | 330 | 7 | 2% |
| Upper arm(s) | 421 | 6 | 2% |
| Neck and back | 84 | 5 | 1% |
| Upper and lower limb(s) | 87 | 5 | 1% |
| Foot (feet), unspecified | 530 | 5 | 1% |
| Scalp | 112 | 4 | 1% |
| Hand(s) and finger(s) | 448 | 4 | 1% |
| Tooth (teeth) | 1363 | 4 | 1% |
| Chest, except internal location of diseases or disorders | 310 | 3 | <1% |
| Arm(s), unspecified | 420 | 3 | <1% |
| Elbow(s) | 422 | 3 | <1% |
| Thigh(s) | 511 | 3 | <1% |
| Scrotum | 3451 | 3 | <1% |
| Neck, except internal location of diseases or disorders | 20 | 2 | <1% |
| Skull | 113 | 2 | <1% |
| Cheek(s) | 134 | 2 | <1% |
| Multiple back regions | 328 | 2 | <1% |
| Multiple body parts, n.e.c. | 899 | 2 | <1% |
| Nose, except internal location of diseases or disorders | 1330 | 2 | <1% |
| Elbow(s) and arms(s) | 4281 | 2 | <1% |
| All other BP Codes with one injury only* | **** | 17 | 5% |
| **Total** | **All** | **342** | **100%** |

*OIICS codes that were only assigned to one injury were combined

**Supplemental Table 3: Top Occupational Injury and Illness Classification System (OIICS) Nature for Commercial Fisherman**

| **Nature Title** | **Nature** | **Freq.** | **Percent** |
| --- | --- | --- | --- |
| Cuts, lacerations | 132 | 65 | 19% |
| Strains | 1233 | 48 | 14% |
| Fractures | 111 | 43 | 13% |
| Sprains, strains, tears, unspecified | 1230 | 35 | 10% |
| Bruises, contusions | 143 | 33 | 10% |
| Sprains | 1232 | 26 | 8% |
| Puncture wounds, except gunshot wounds | 133 | 13 | 4% |
| Concussions | 162 | 13 | 4% |
| Swelling, inflammation, irritation—n.e.c. | 1973 | 8 | 2% |
| Abrasions, scratches | 1979 | 7 | 2% |
| Nonspecified injuries and disorders, n.e.c. | 141 | 6 | 2% |
| Crushing injuries | 1971 | 5 | 1% |
| Drownings | 192 | 4 | 1% |
| Dislocation of joints | 1212 | 4 | 1% |
| Major tears to muscles, tendons, ligaments | 1231 | 4 | 1% |
| Amputations | 1311 | 4 | 1% |
| Soreness, pain, hurt--nonspecified in.. | 1972 | 4 | 1% |
| Avulsions, enucleations | 1312 | 2 | <1% |
| Heat (thermal) burns, unspecified | 1520 | 2 | <1% |
| All Other Nature Codes with one injury only* | **** | 16 | 5% |
| **Total** | **All** | **342** | **100%** |

*OIICS codes that were only assigned to one injury were combined

**Supplemental Table 4: Top Occupational Injury and Illness Classification System (OIICS) Body Part (2-digit level) for Seafood Processors**

| **Body Part (BP) Title** | **Body Part** | **Freq.** | **Percent** |
| --- | --- | --- | --- |
| Hand(s) | 44 | 216 | 26% |
| Back, including spine, spinal cord | 32 | 88 | 11% |
| Leg(s) | 51 | 69 | 8% |
| Shoulder(s), including clavicle(s), scapula(e) | 41 | 64 | 8% |
| Face | 13 | 63 | 8% |
| Foot (feet) | 53 | 54 | 6% |
| Wrist(s) | 43 | 47 | 6% |
| Ankle(s) | 52 | 42 | 5% |
| Arm(s) | 42 | 39 | 5% |
| Abdomen | 33 | 24 | 3% |
| Pelvic region | 34 | 18 | 2% |
| Multiple upper extremities locations | 48 | 16 | 2% |
| Chest, including ribs, internal organs | 31 | 15 | 2% |
| Head, unspecified | 10 | 14 | 2% |
| Other multiple body parts | 89 | 14 | 2% |
| Multiple body parts, unspecified | 80 | 9 | 1% |
| Cranial region, including skull | 11 | 8 | 1% |
| Neck, except internal location of diseases or disorders | 20 | 6 | <1% |
| Body System | 6 | 6 | <1% |
| Multiple trunk locations | 38 | 5 | <1% |
| Multiple lower extremities locations | 58 | 3 | <1% |
| Neck and back | 84 | 3 | <1% |
| Unknown | Missing | 2 | <1% |
| Ear(s) | 12 | 2 | <1% |
| Multiple head locations | 18 | 2 | <1% |
| **Total** | **All** | **829** | **100%** |

**Supplemental Table 5: Top Occupational Injury and Illness Classification System (OIICS) Nature for Seafood Processors**

| **Nature Code** | **Nature** | **Freq.** | **Percent** |
| --- | --- | --- | --- |
| Sprains, Strains, tears, unspecified | 1230 | 317 | 38% |
| Bruises, contusions | 143 | 179 | 22% |
| Cuts, lacerations | 132 | 93 | 11% |
| Nonspecified injuries and disorders, n.e.c. | 1979 | 85 | 10% |
| Fractures | 111 | 37 | 4% |
| Puncture wounds, except gunshot wounds | 133 | 17 | 2% |
| Dermatitis and reactions affecting the skins--acute, unspecified | 1950 | 12 | 1% |
| Strains | 1233 | 10 | 1% |
| Dislocations, unspecified | 1210 | 9 | 1% |
| Hernias due to traumatic incidents | 124 | 8 | 1% |
| Abrasions, scratches | 141 | 7 | <1% |
| Poisoning, toxic, noxious, or allergenic effect, unspecified | 1960 | 7 | <1% |
| Concussions | 162 | 6 | <1% |
| Swelling, inflammation, irritation, nonspecified injury | 1973 | 5 | <1% |
| Surface wounds and bruises, n.e.c. | 149 | 4 | <1% |
| Heat (thermal) burns, unspecified | 1520 | 4 | <1% |
| Blisters | 142 | 3 | <1% |
| Chemical burns and corrosions, unspecified | 1510 | 3 | <1% |
| First degree heat (thermal) burns | 1521 | 3 | <1% |
| Other burns, unspecified | 1590 | 3 | <1% |
| Effects of reduced temperature, unspecified | 1710 | 3 | <1% |
| Surface wounds and bruises, unspecified | 140 | 2 | <1% |
| Sprains | 1232 | 2 | <1% |
| Amputations, avulsions, enucleations, unspecified | 1310 | 2 | <1% |
| All other Nature codes with only one injury* | **** | 8 | 1% |
| **Total** | **All** | **829** | **100%** |

*OIICS codes that were only assigned to one injury were combined
